# Human Melanoma Cells Over-Express Extracellular Matrix 1 (ECM1) Which Is Regulated by TFAP2C

**DOI:** 10.1371/journal.pone.0073953

**Published:** 2013-09-02

**Authors:** Geeta Lal, Piedad Gomez Contreras, Mikhail Kulak, George Woodfield, Thomas Bair, Frederick E. Domann, Ronald J. Weigel

**Affiliations:** 1 Department of Surgery, University of Iowa, Iowa City, Iowa, United States of America; 2 Iowa Institute of Human Genetics, University of Iowa, Iowa City, Iowa, United States of America; 3 Department of Free Radical and Radiation Biology, University of Iowa, Iowa City, Iowa, United States of America; 4 Department of Biochemistry, University of Iowa, Iowa City, Iowa, United States of America; National Center for Scientific Research Demokritos, Greece

## Abstract

Extracellular matrix 1 (ECM1) is over-expressed in multiple epithelial malignancies. However, knowledge regarding the expression of ECM1 in melanomas and the mechanisms of ECM1 regulation is limited. In this study, we found that ECM1 is over-expressed in several melanoma cell lines, when compared to primary melanocytes, and furthermore, that ECM1 expression paralleled that of TFAP2C levels in multiple cell lines. Knockdown of TFAP2C in the A375 cell line with siRNA led to a reduction in ECM1 expression, and upregulation of TFAP2C with adenoviral vectors in the WM793 cell line resulted in ECM1 upregulation. Utilizing 5’ RACE to identify transcription start sites (TSS) and luciferase reporter assays in the ECM1-overexpressing A375 cell line, we identified the minimal promoter region of human ECM1 and demonstrate that an approximately 100bp fragment upstream of the TSS containing a TATA box and binding sites for AP1, SP1 and Ets is sufficient for promoter activity. Chromatin immunoprecipitation and direct sequencing (ChIP-seq) for TFAP2C in the A375 cell line identified an AP2 regulatory region in the promoter of the ECM1 gene. Gelshift assays further confirmed binding of TFAP2C to this site. ECM1 knockdown reduces melanoma cell attachment and is consistent with findings that ECM1 overexpression has been associated with a poor prognosis. Our investigations show an as yet unrecognized role for TFAP2C in melanoma via its regulation of ECM1.

## Introduction

Melanoma is the sixth most common cancer in the United States and survival is contingent upon the clinical stage at diagnosis. The cumulative 5-year survival among individuals diagnosed with melanoma localized to the skin is in excess of 90% compared to the 64% and 16% 5-year survival observed for those diagnosed with regional and distant metastases, respectively. Transcription factor Activated Protein 1 (TFAP2) expression has been implicated as an important prognostic indicator in melanoma. Specifically, loss of TFAP2A expression is associated with progression of human melanoma [1]. Studies also show that inactivation of TFAP2A via a dominant negative mechanism augments the tumorigenicity of non-metastatic melanoma cell lines in nude mice.

The human Extracellular Matrix 1 (*ECM1*) gene maps to 1q21, contains 10 exons and encodes an 85 kDa protein [2–5]. *ECM1* over-expression has been reported in several malignancies, including invasive ductal breast carcinomas, esophageal squamous cell carcinomas, gastric cancers, colorectal, lung and thyroid cancers [6–9]. Overall, the average frequency of ECM1 positivity in carcinomas was 75%, which is much higher than that of epithelial hyperplasia (11.7%) or normal tissues (7.1%) [6]. These results suggested that although ECM1 may not be a tumor specific protein, its expression levels were significantly elevated in malignant epithelial tumors.

ECM1 also appears to have prognostic significance in several malignancies. Wang et al. [6] showed that breast and lung tumors with lymph node metastases were more likely to be ECM1- positive than those without lymph node metastases. Our group has previously completed a single-institution study which showed that *ECM1* over-expression confers a worse long-term prognosis, independent of other prognostic markers, in patients with invasive breast carcinomas [10]. Increased ECM1 expression has also been noted in laryngeal carcinomas, hepatocellular carcinomas and cholangiocarcinomas where it was correlated with microvessel density, growth, metastasis and poor prognosis [11–13]. In addition, some studies suggest *ECM1* expression may be incorporated into a scoring model to predict high-risk thyroid cancers [8].

Although ECM1 expression may have prognostic significance, not much is known about the regulation of ECM1 expression or the mechanisms of its effect on tumor prognosis. With respect to *ECM1* regulatory pathways, cDNA microarray analysis of the downstream effects of over-expressing a constitutively active β-catenin have identified ECM1 as a putative target gene of the activated Wnt/β-catenin signal transduction pathway [14]. Additional studies in human breast epithelial cells demonstrate that over-expression of TFAP2A or TFAP2C induces *ECM1* expression [15], suggesting that ECM1 expression may be regulated by TFAP2. ECM1 has been reported to play a role in promoting angiogenesis and ECM1 expression appears to correlate with rapid cell division [11].

The status of ECM1 expression is melanomas has not been previously examined. We hypothesized that ECM1 is over-expressed in melanoma cell lines and furthermore, based on the discussion above, that ECM1 expression is regulated by TFAP2. In this paper, we have identified the minimum promoter region of the human ECM1 gene and demonstrated regulation of ECM1 by TFAP2C.

## Materials and Methods

### Cell lines and culture

Melanoma cell lines studied included A375, M21, M14 (subclone 2C5), WM793 and melanocytes. The cells were purchased from ATCC, Manassas, Virginia (A375, M14, WM793, melanocytes), and a generous gift from Dr. Kris DeMali (M21) at the University of Iowa [16]. Cells were maintained in RPMI-1640 (with 10% Fetal Bovine Serum (FBS), penicillin, 100U/ml and Streptomycin 100mg/ml) and incubated at 37^0^C in a humidified atmosphere (5% CO_2_). Melanocytes were maintained in culture using complete growth medium consisting of Minimum Essential Medium Eagle (MCDB) 153 supplemented with 4% fetal bovine serum, insulin (5 µg/ml), α-tocopherol (1 µg/ml), 1% penicillin/streptomycin/amphotericin, human basic fibroblast growth factor (bFGF, 0.6 ng/ml), phorbol 12-myristate 13-acetate (PMA, 8 nM), and bovine pituitary extract (BPE, 13 µg/ml). All the medium components were from Sigma (Sigma Aldrich, St. Louis, MO, USA) except for BPE (Clonetics, San Diego, CA, USA). Cells were harvested for RNA and protein extraction when they were 80-90% confluent using EDTA 0.6mM/PBS solution for detaching.

### cDNA preparation and Real time RT-PCR

Total RNA was extracted after homogenization of cells and tissues using RNeasy mini kit (Qiagen Sciences, Maryland MD) and performing DNase digestion (RNase free DNase set, (Qiagen, Valencia CA)) during the RNA extraction. Total RNA (1 µg) was reverse transcribed with the High Capacity cDNA Reverse Transcription Kit (Applied Biosystems, Foster City CA). The cDNA reaction was diluted to 1:10 for use as template for real-time RT-PCR.

TaqMan Gene Expression Assays primers and probes specific to TFAP2A, TFAP2C and ECM1(Hs01029413_m, Hs00231476_m and Hs00189435_m respectively) were used for expression analyses and 18S ribosomal primers and probes (Applied Biosystems, Foster City, CA) were used as internal controls. PCR amplifications were performed in a final reaction volume of 10 µl containing, 5.5 µl of TaqMan Universal PCR Master Mix (Applied Biosystems, Foster City, CA), 0.5 µl of the primers and probes mix and 4.5 µg of the cDNA diluted solution. The cycling conditions were as follows: one cycle of 2 minutes at 50^0^C, one cycle of 10 minutes at 95^0^C, 40 cycles of denaturation (15 seconds at 95^0^C) and annealing/extension (1 minute at 60^0^C). All reactions were carried out in the Step 1 Plus Real-Time PCR Systems Thermocycler (Applied Biosystems, Foster city, CA). All quantitative PCR reactions were carried out in triplicate and repeated at least twice. The ΔCt for mRNA expression was calculated relative to the Ct (threshold cycle) of 18S ribosomal RNA. Relative mRNA expression was calculated using the formula 2 ^(-ΔΔCt)^.

### Western blotting

Nuclear and Cytoplasmic extracts (NE-PER, Pierce Biotechnology, Rockford, IL) were quantified by BCA Protein Assay (Pierce/Thermo Scientific, Rockford IL) and 10 µg per sample were denatured, reduced and loaded onto10% NuPAGE-SDS electrophoresis gels (Invitrogen, Carlsbad, CA) and blotted to nitrocellulose membrane (GE Healthcare, Piscataway, NJ). After blocking was accomplished with 5% milk Tris Buffer Saline Tween-20 solution, the primary antibody was added - TFAP2 (Abcam, Cambridge MA), to the nuclear fraction membrane at 1:15,000 dilution or ECM1 antibody (Santa Cruz Polyclonal anti ECM-1 (N-17), cat No sc-65086), 1:1000 to the cytoplasmic fraction. HRP-secondary antibody was used and the membranes developed using Enhanced Chemiluminescence Plus Western Blotting Kit (GE Healthcare, Piscataway, NJ). GAPDH and β-actin expression were used as loading controls.

### siRNA knockdown

*TFAP2 and ECM1* expression was knocked down using siGenome siRNA (Thermo Scientific/Dharmacon, Lafayette, CO) and ECM1 Silencer Select Pre-deigned siRNA (Ambion, Carlsbad CA), respectively. Silencer Select Negative Control 1 (Ambion, Carlsbad CA) was used as well. Approximately 100,000 cells were seeded to 6-well culture plates and grown to around 60% confluence. The oligonucleotides were diluted in 50 µL OptiMEM I medium (Gibco). Lipofectamine RNAiMAX (Invitrogen, Carlsbad CA) was used as the transfection agent. The cells were incubated at 37°C in a CO_2_ incubator for up to 96 hours.

### Expression vectors

Approximately 300,000 cells/well were seeded in 12-well plates and Adenovirus Ad5CMVUI9988-AP2-gamma-1 or Ad5CMVUI9988-AP2-alpha-1 from University of Iowa, Central Vector Core were added to 0.5 ml of plain media at 150 to 300 MOI (multiplicity of infection). Ad5CMVhrGFP was used as control. The virus suspension was incubated in culture media for 20 minutes and added to the plates. The cells were incubated at 37^o^C for 4 hours after which culture media with 10% FBS and antibiotics was added to reach a final volume of 1 ml. The cells were incubated up to 96 hours.

### 5’ RACE (Rapid Amplification of cDNA Ends), creation of deletion constructs, transient transfections and reporter gene assay

Identification of the mRNA transcription start sites for *ECM1* were examined in the A375 cell using a 5’-RACE system for rapid amplification of cDNA ends (Version 2.0, Life Technologies) according to the manufacturer’s protocol and using previously described primers [3] and cycling conditions. Subsequently, an approximately 2 kb fragment including sequences upstream and approximately 100 bp downstream of the transcription start site was directionally cloned into the pGL3 basic expression vector (Promega, Madison, WI) for firefly luciferase. Briefly, competent *E. coli* cells were transformed with the plasmids and then plated out on LB-Amp agar. Individual colonies were grown in LB-Amp liquid cultures. Plasmid mini-preps were then carried out. Recombinant plasmids were identified after digestion with BglIII and NheI and gel electrophoresis. DNA was sequenced to confirm the identity of the insert and its direction. Once the full length fragment was cloned, deletion plasmid constructs were cloned in a similar fashion, by generating successively smaller PCR products.

The full-length fragment and deletion constructs were examined. Briefly, the plasmids were transfected using TransFast Transfection Reagent (Promega, Madison, WI) into the A375 cell line according to the manufacturer’s instructions and promoter activity was assessed by the Dual Luciferase Reporter Assay System (Promega, Madison, WI). For this, 2 x 10^5^ cells were seeded in 12 well plates in 1 ml of medium without antibiotics to 80% confluence. Next, 500 ng of pGL3-promoter construct and 150 ng of pRL-SV40 plasmid (positive control vector with *Renilla* luciferase gene and SV40 promoter) were added to 6 replicate wells, and 500 ng of pGL3-basic vector (without insert) with 150 ng pRL-SV40 were added to 6 replicate wells (to determine background luciferase activity). Then, each was combined with 6 µl of Transfection Reagent in a total volume of 500 µl culture media without antibiotics and FBS. Cells were incubated for 48 hrs at 37°C, following which the medium removed and wells washed gently in PBS. 250 µl of passive lysis buffer (Promega) was added to each well, and after rocking for 15 minutes, the supernatants were transferred to a tube and centrifuged. Next, 20 µl of lysate was transferred to a tube containing 100 µl of Luciferase Assay Reagent II and firefly luciferase activity was measured using a TD-20/20 luminometer (Turner Designs). Following this, Stop & Glo reagent was added, and Renilla luciferase activity was measured. Triplicates of each cell line/plasmid transfection combination were used to calculate the mean luciferase activities for each. Luciferase activity was normalized to that seen in the PGL3 basic vector and to Renilla luciferase activity (as a measure of transfection efficiency).

### Site directed mutagenesis

Mutations of the putative TFAP2 and other transcription factor binding sites were introduced in the constructs by PCR with the QUIKChange II XL Site-directed mutagenesis kit, according to the manufacturers protocols (Stratagene, Agilent technologies, Santa Clara, CA). Mutations were verified by direct sequencing.

### Chromatin Immunoprecipitation with Direct Sequencing (ChIP-seq)

Preparation of DNA for these experiments were carried out as previously described with minor modifications [17]. Briefly, one hundred million cells were cross-linked for 10 min at 37°C using 0.7% formaldehyde, 0.125 M glycine. Cells were washed twice with PBS, resuspended in lysis buffer [50 mmol/L HEPES (pH 8.0), 85 mmol/L KCl, 0.5% IPEGAL CA-630 + Roche complete protease inhibitors (Indianapolis, IN)] and incubated for 5 min. Cells were collected by centrifugation and cell pellets were frozen in liquid nitrogen and stored at minus 80°C. Cell pellets were thawed in lysis buffer, collected by centrifugation and cell nuclei were resuspended in RIPA buffer [1xPBS, 1% IPEGAL CA-630, 0.5% sodium deoxycholate, 0.1% SDS + Roche Complete protease inhibitors]. Chromatin was sonicated using conditions determined empirically for A375 cells to achieve an optimal fragment length between 400 to 100 bp. After sonication, samples were centrifuged at 20,000 × g for 10 min at 4°C. The supernatant containing cross-linked DNA/histones was diluted with IP dilution buffer to 2 mg/ml [0.01% SDS, 1.1% Triton-X 100, 1.2 mmol/L EDTA, 16.7 mmol/L Tris-Cl, pH 8.1, 167 mmol/L NaCl] plus protease inhibitors]. Half the sample was immunoprecipitated with 10 µg of TFAP2C monoclonal antibody SC-12762X (Santa Cruz Biotechnology, Santa Cruz, CA), and half with control nonspecific IgG (Upstate, Waltham, MA) with the addition of Dynal sheep anti-mouse Dynabeads and allowed to recognize their antigens overnight at 4°C with rotation. Protein/antibody/DNA complexes were collected magnetically followed by washing and elution. Protein/DNA cross-links were reversed using 200 mmol/L NaCl at 65°C overnight. DNA was treated with Proteinase K and RNase A and was recovered with Qia Quick PCR Kit (Qiagen) according to manufacturer’s suggested protocol. Input chromatin was processed identically as the IP chromatin samples. Purified DNA was quantified by using a NanoDrop ND-1000 (NanoDrop, Wilmington, DE).

Chromatin-immunoprecipitated DNA samples were prepared for and sequenced using an Illumina Genome Analyzer GAII at the Iowa State University DNA Facility according to the instructions of the manufacturer (Illumina, San Diego, CA). Chip-seq libraries were prepared by repairing the DNA fragment ends, adding an “A” to the 3′ end of the repaired fragments, and ligating adapters using Illumina’s ChIP-Seq sample prep kit. Libraries were enriched by 15 cycles of PCR amplification and size selected (200–300 bp) on a 2% agarose gel. The library was quantified and the sizes checked on a Bioanalyzer 2100 using the DNA 1000 and high sensitivity DNA chips. The library was clustered on the flow cell using the v.2 cluster generation kit (Illumina). The flowcell was loaded onto a Genome Analyzer II and subjected to single sequencing using v.3 36 cycle sequencing kit (Illumina). Image analysis, base calling and alignment were performed using Pipeline v.1.3 software.

### Analysis of ChIP-Seq Data

Illumina reads were aligned using bow tie version 0.12.7 to hg18. BEDTools version 2.11 was used to generate a bedgraph file showing coverages, beddGraphToBigWig was used to generate a bigwig file that was subsequently uploaded and viewed in the UCSC genome browser. The area upstream of ECM1 was visualized and the extents of the most prominent peak manually specified. The data discussed in this publication have been deposited in NCBI’s Gene Expression Omnibus [18] and are accessible through GEO Series accession GSE41234

### Gel Shift Assay

This was performed using Gel Shift Assay Core System Cat# E3050 (Promega). Labeling of the 140bp probe (NCBI36/hg18 chr1:148747047-148747187) was performed as recommended in the manufacturer’s protocol. TFAP2C protein was generated from pcDNA3.1-AP2C [19] using TNT T7 Quick Coupled Transcription/Translation System Cat#L1170 (Promega). 2 µg Abs AP-2C (6E4/4) (Santa Cruz) were added per reaction as needed.

### Functional studies

Migration was evaluated in the A375 and M21 cell lines with the wound assay performed 24 hrs after silencing. The cell monolayer was scratched with a 200 µl pipette tip to create a wound. Plates were washed to remove floating cells and were incubated with D-MEM (supplemented). Migrating cells from the edge of the wound were photographed at various time points. Percentage of open/healed wound area was calculated using the T-Scratch software [20]. Cell invasion assays were performed with BD BioCoat™ Matrigel™ Invasion Chamber (BD Biosciences, Bedford, MA) following the manufacturer’s directions. Briefly, cells were detached from the culture plates using 0.6mM EDTA, washed and re-suspended in D-MEM culture media without supplements. A total of 0.5 ml of cell suspension at 500,000 cells/ml were added to each insert and incubated for 24 hrs, at 37°C, 5% CO_2_. Non-invading cells were cleaned from the upper surface of the insert by scrubbing with a cotton swab, following which the inserts were incubated with 0.5ml of Thiazolyl Blue Tetrazolium Bromide (MTT) solution (Sigma-Aldrich, Saint Louis, MO) at 0.5mg/ml dissolved in culture media without supplements and phenol red for 1 hr at 37°C. After staining, the membranes were cut off from the inserts, and MTT dye was dissolved with dimethyl sulfoxide and absorbance measured at 550nm. Cell adhesion was examined as follows. Cell supernatant was discarded and cells were detached with 0.6mM EDTA, centrifuged and resuspended in complete culture media. Approximately 50,000 cells were seeded per well in a 12 well plate in Triplicate. The cells were incubated at 37^o^C, 5% CO^2^ for 2 hours to allow the cells to attach to the bottom of the plate. The supernatant was discarded and the cells were stained with warm MTT solution at 0.5 mg/ml in RPMI-1640. Then 250 µL of Dimethyl Sulfoxide (DMSO), was added per well and the cells were transferred to a 96 well plate with 100 µl DMSO colored solution per well. Absorbance was read at 550 nm.

## Results

### ECM1 expression in melanoma cells was correlated with TFAP2C expression Primary

**Figure 1:**
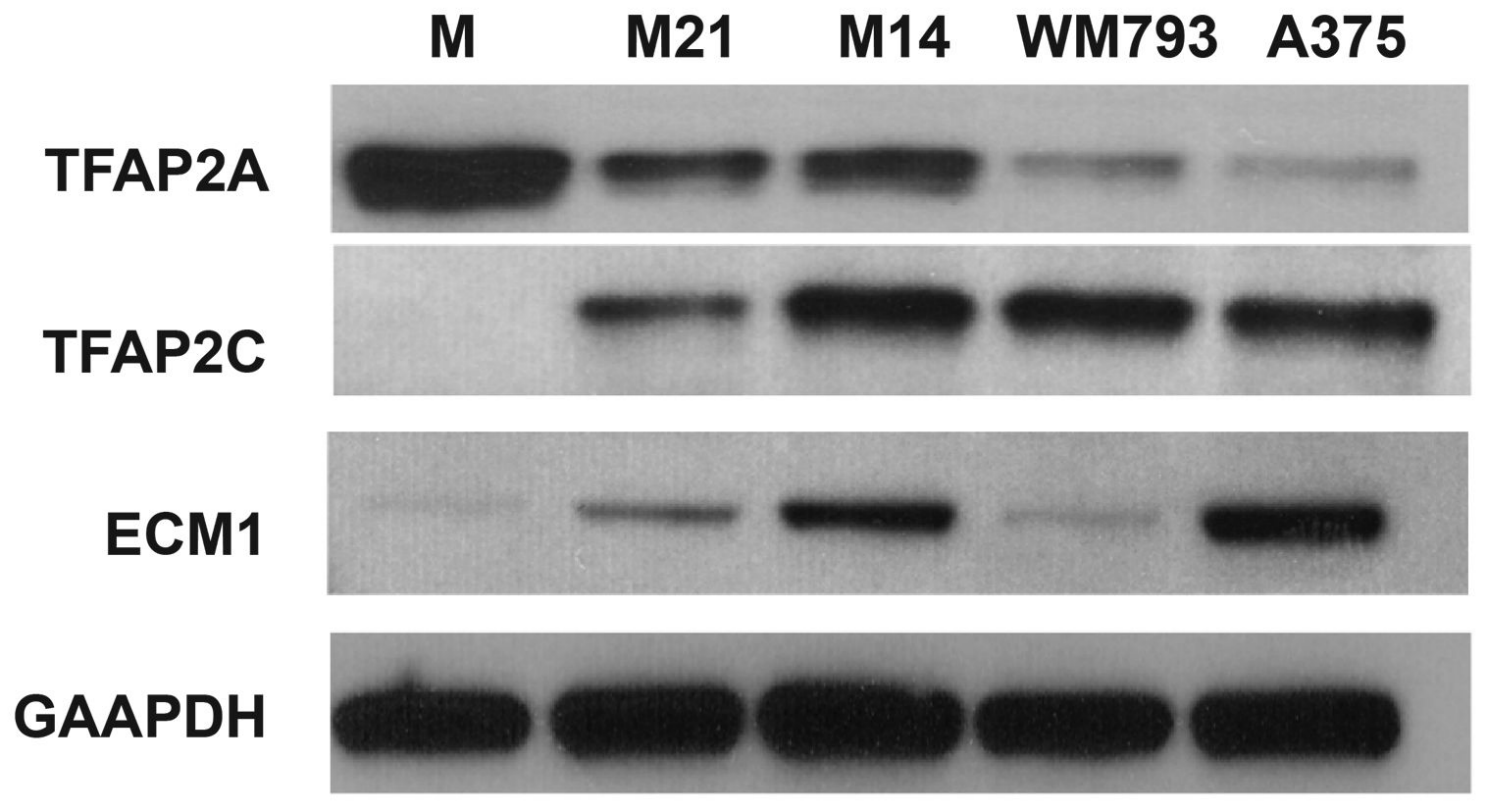
ECM1 expression in melanoma cell lines. Western blots showing expression of TFAP2A, TFAP2C and ECM1 in primary melanocytes (M) and melanoma cell lines (M21, M14 subclone 2C5, WM793 and A375). GAPDH was used as a loading control.

melanocytes and melanoma cell lines were screened for ECM1 expression. Since previous studies in HMECs had shown that both TFAP2A and TFAP2C induce ECM1, expression of these transcription factors was also examined. As shown in Figure **1**, primary melanocytes highly expressed TFAP2A but demonstrated minimal expression of TFAP2C and ECM1. In contrast, the A375 cell line had minimal TFAP2A expression but overexpressed both TFAP2C and ECM1. Examination of the other cell lines (M21, M14 and WM793) also demonstrated an association between TFAP2C and ECM1 expression.

### Altering TFAP2 expression affects ECM1 expression

**Figure 2:**
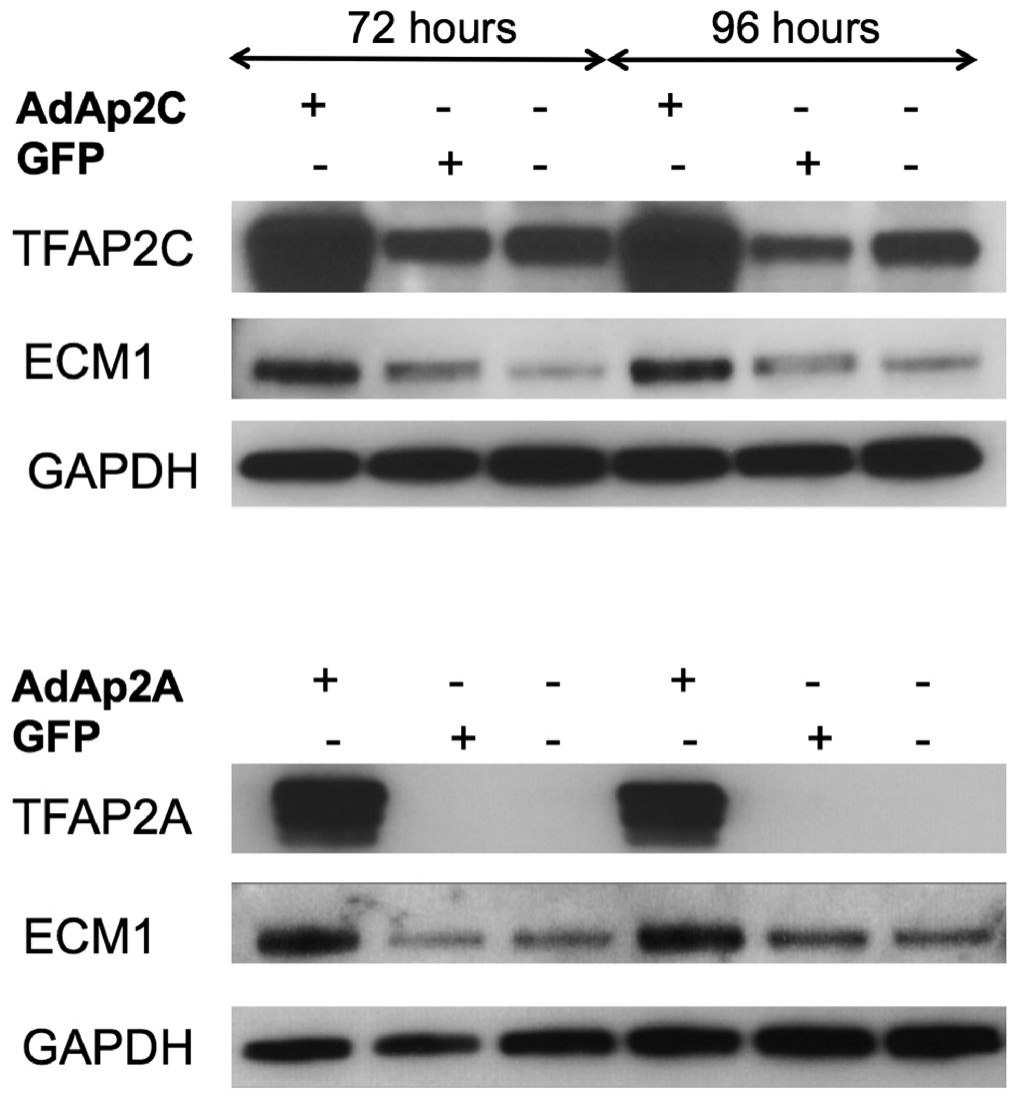
Regulation of ECM1 expression in WM793B. Upregulation of TFAP2 in WM793B melanoma cells using AdAP2A andAdAP2C leads to increased ECM1 expression compared to mock infected cells and those infected with GFP vector (used as an infection control) as determined by Western blot.

The A375 and WM793B cell lines were used to further evaluate the effects of TFAP2 on ECM1 expression. Recombinant adenovirus vectors were used to upregulate TFAP2A and TFAP2C in the WM793B cell line As shown in Figure **2** overexpression of the transcription factors was accompanied by upregulation of ECM1 protein at 72 and 96 hours.

**Figure 3:**
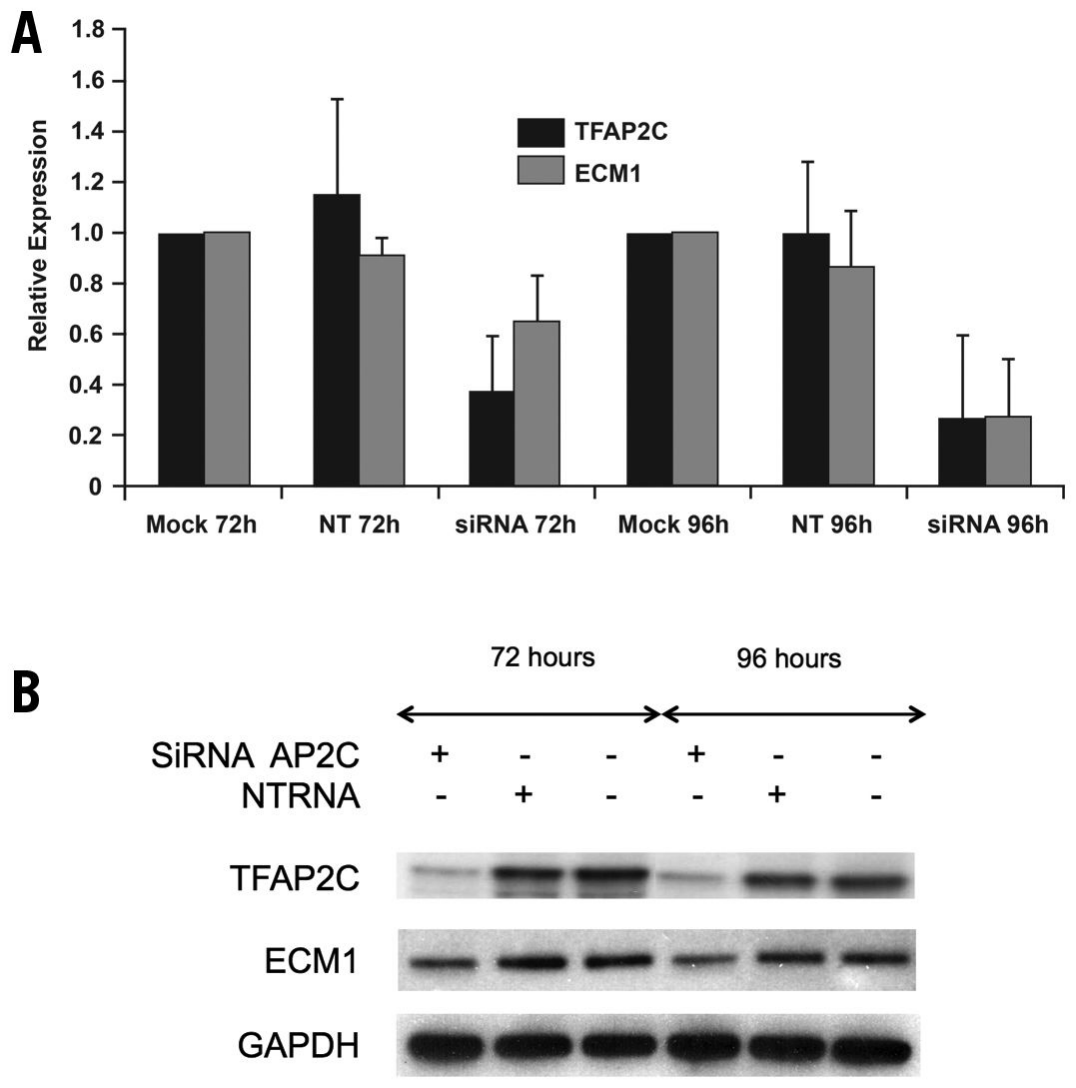
Regulation of ECM1 in A375. Blocking of TFAP2C expression using siRNA in A375 cell line leads to reduced ECM1 mRNA by 72 hours (A) and reduced ECM1protein expression by 96 hours (B). Non-targeting (NT) siRNA was used as a transfection control. Samples were run in triplicate and the graph represents the mean results from 2 different experiments. Expression levels are relative to those in mock-treated A375 cells.

Since the role of TFAP2C is relatively understudied compared to TFAP2A, we elected to further study the effects of modulating TFAP2C expression. TFAP2C was knocked down in the A375 cell line using siRNA. At 72 hours, TFAP2C mRNA expression was reduced by > 60% (mean of 2 separate experiments) in cells transfected with siRNA to TFAP2C (Figure **3A**). TFAP2C knockdown was associated with a concomitant reduction in ECM1 expression > 70% by 96 hours. Knockdown of TFAP2C was also seen at the protein level (Figure **3B**), and was accompanied by a 25-35% decrease in ECM1 (as calculated by densitometry, data not shown)

### 5’ RACE indicates a major and minor transcription start site for ECM1

**Figure 4:**
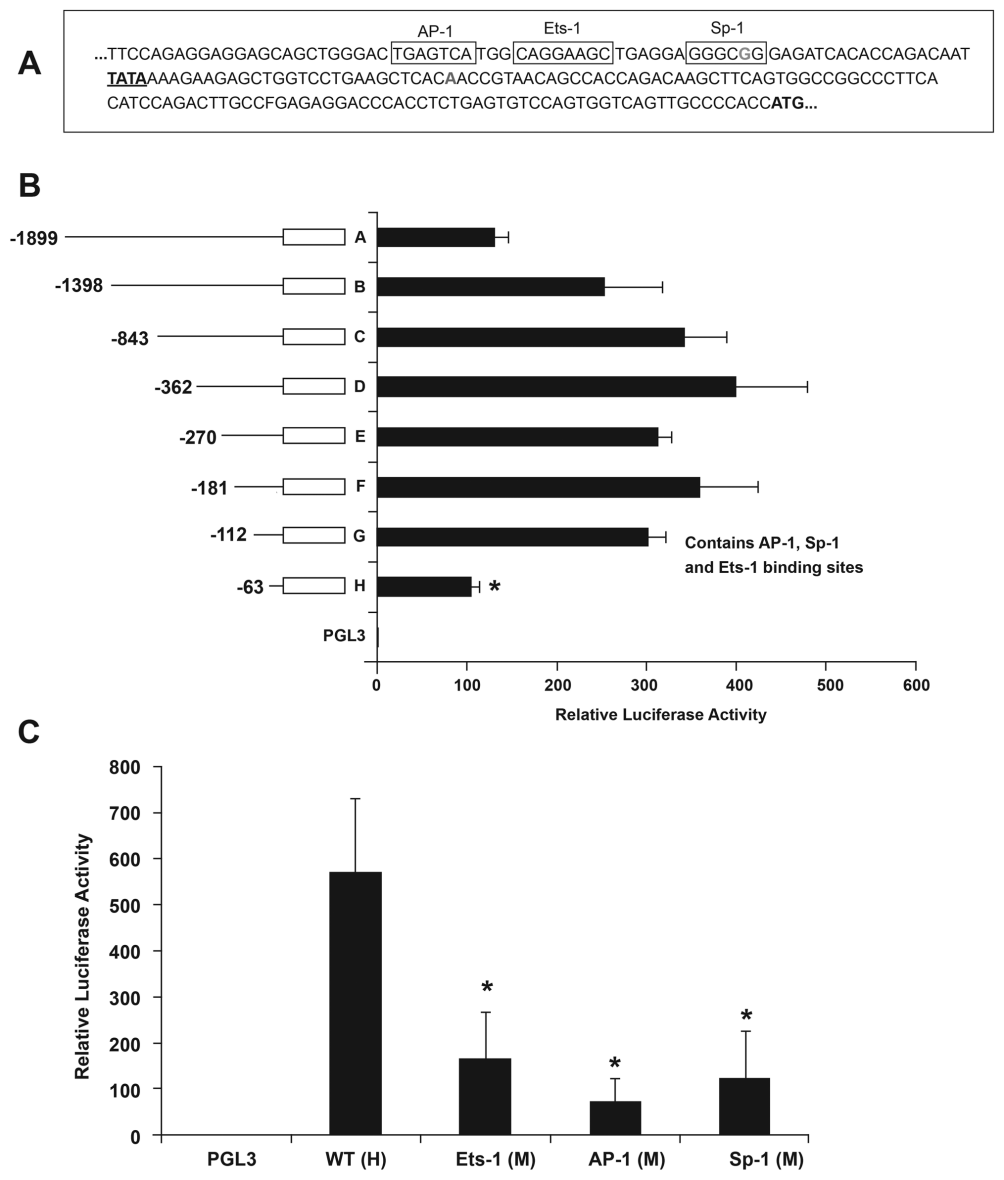
5’ RACE in the A375 cell line. Sequence upstream of the ECM1 start site showing a major (red) and minor (green) transcription start site (TSS), upstream of the start codon ATG. A TATA box is seen upstream of the major transcription start site. Boxes represent transcription factor binding sites (**A**). Dual Luciferase assay in A375: BFigure shows Relative Luciferase activity (run in triplicates, results shown as mean and SD) of fragments containing sequences upstream of the ECM1 TSS. There is a significant (*, p=0.0005) drop in luciferase activity when fragment G is further deleted (**B**). Site directed mutagenesis: Luciferase activity of Fragment H mutated (designated as M) at the Ets, AP-1 and Sp-1 sites is significantly lower than that observed for the WT construct * p < 0.05. The transfections were done in triplicate and the graph represents the mean value of the 6 values obtained for each plasmid (**C**).

In order to define the regulatory regions of ECM1, 5’ RACE was used to identify the mRNA transcription start sites in the A375 cell line A major transcription start site (8/10 clones) was identified at 101 bp and a minor transcription start site was noted at 151 bp (2/10 clones) upstream of the start codon ATG. A TATA box was present 30 bp upstream of the major transcription start site, as shown in Figure **4A**. Luciferase reporter assays of the full length and deletion constructs were examined in the ECM1-positive A375 cell line. The constructs contained the following base pairs (labeled in relation to the TSS) as verified by direct sequencing: A (-1899 to +99), B (-1398 to +99), C (-843 to +99), D (-362 to +99), E (-270 to +99), F (-181 to +99), G (-112 to + 99) and H (-63 to +99). As shown in Figure **4B**, the full-length fragment A had detectable luciferase activity indicating that a regulatory region is present in this construct. Highest luciferase activity was noted in fragment D. Luciferase activity of the larger constructs was lower, suggesting possible inhibitory or repressor effects in this region. Luciferase activity remained at a similar level for fragments E, F and G, but was significantly reduced (p= 0.0005) when the construct was further deleted resulting in fragment H (containing sequences from -64 to +99). Fragment G (containing sequences from -113 to +99) also includes conserved binding sites for AP1, Sp1 and Ets1, similar to those seen in the mouse Ecm1 promoter [21]. Point mutations of these sites were generated by site directed mutagenesis in fragment G (data not shown) and promoter activities of the mutant fragments were compared with those of the WT constructs. Each mutation resulted in a significant reduction of promoter activity (p = 0.0006 for Ets-1, p=0.0004 for AP-1 and p=0.0003 for Sp-1 respectively), indicating that these sites are also essential for ECM1 expression in human cells (Figure **4C**).

### ChIP-seq identified potential TFAP2C binding sites in the ECM1 promoter

**Figure 5:**
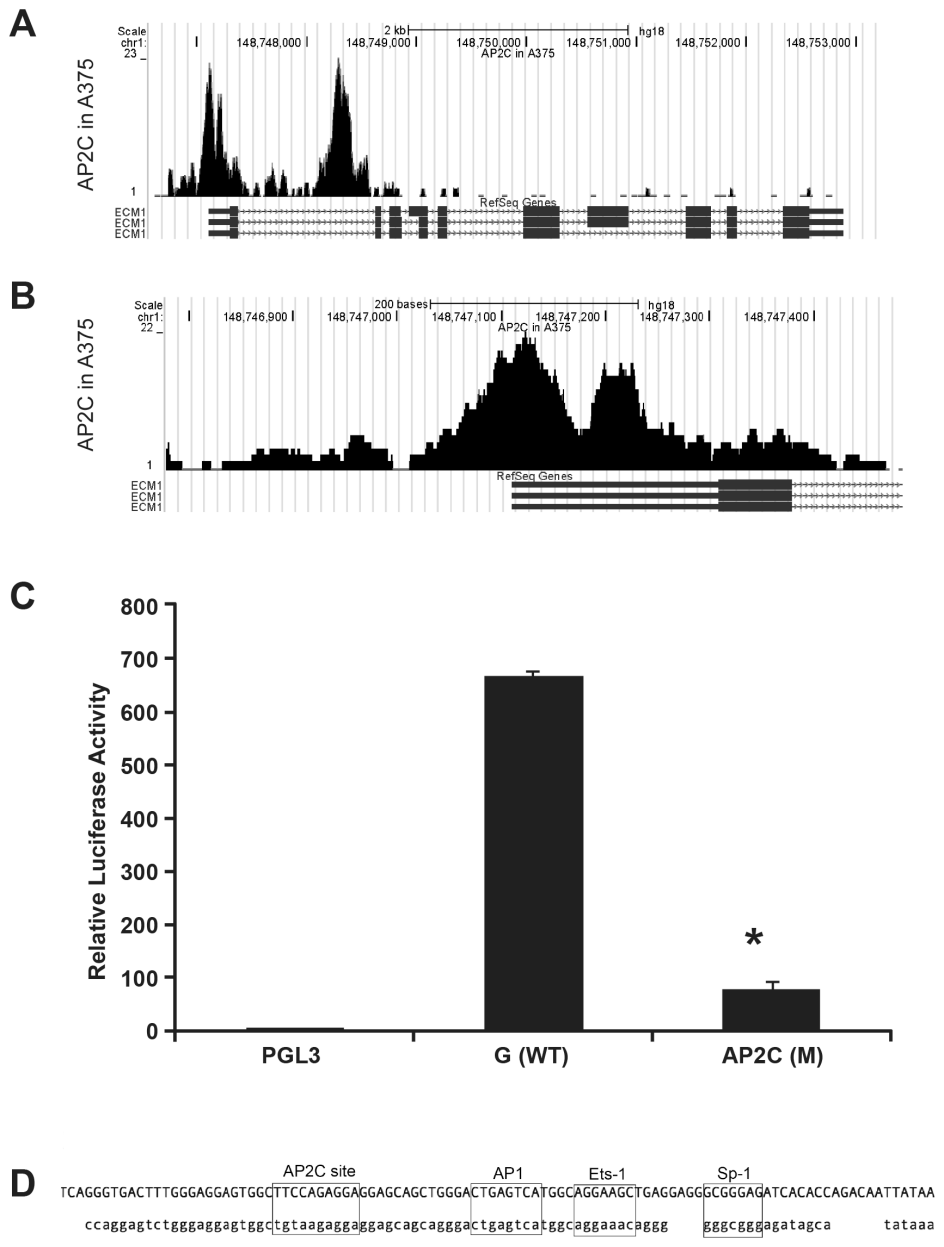
AP2C Chip-seq data from A375 cells. A global view of the 5’ region of ECM1 (A) and a more honed in view of the promoter region (B). A notable peak was identified centered at chromosomal location 148747123. Reads were aligned with Bow tie and bam files then coverage was determined using the BEDtools version 2.11. The bedgraph output was converted to bigwig and visualized in the UCSC genome browser. Mutating the putative TFAP2C binding site significantly reduced the promoter activity compared to the fragment containing the WT sequence (**C**). Alignment of the human and mouse promoter sequences showing the TFAP2C binding site and those of other transcription factors (**D**).

Multiple putative TFAP2 binding sites are present in an approximate 5000 bp fragment surrounding the ECM1 start site (Transcription Element Search System (TESS)), including some sites which are located within the first intron of ECM1 (data not shown) [22]. In order to determine the most biologically relevant sites in relation to the promoter region identified in our experiments, ChIP-seq was performed in the A375 cell line to identify regions of the genome bound by endogenously expressed TFAP2C. As shown in Figure **5A and 5B**, a significant peak for TFAP2C was centered near a potential TFAP2C binding site at -101 (TCCAGAGG, centered at chromosomal location 148747123) localized within the identified promoter region. Mutating the putative TFAP2C site significantly reduced the promoter activity compared to the fragment containing the WT sequence (p<0.0001, Figure **5C**). The corresponding site in the mouse promoter can be seen in Figure 5D

### EMSA showed that TFAP2C directly binds to the ECM1 promoter

**Figure 6:**
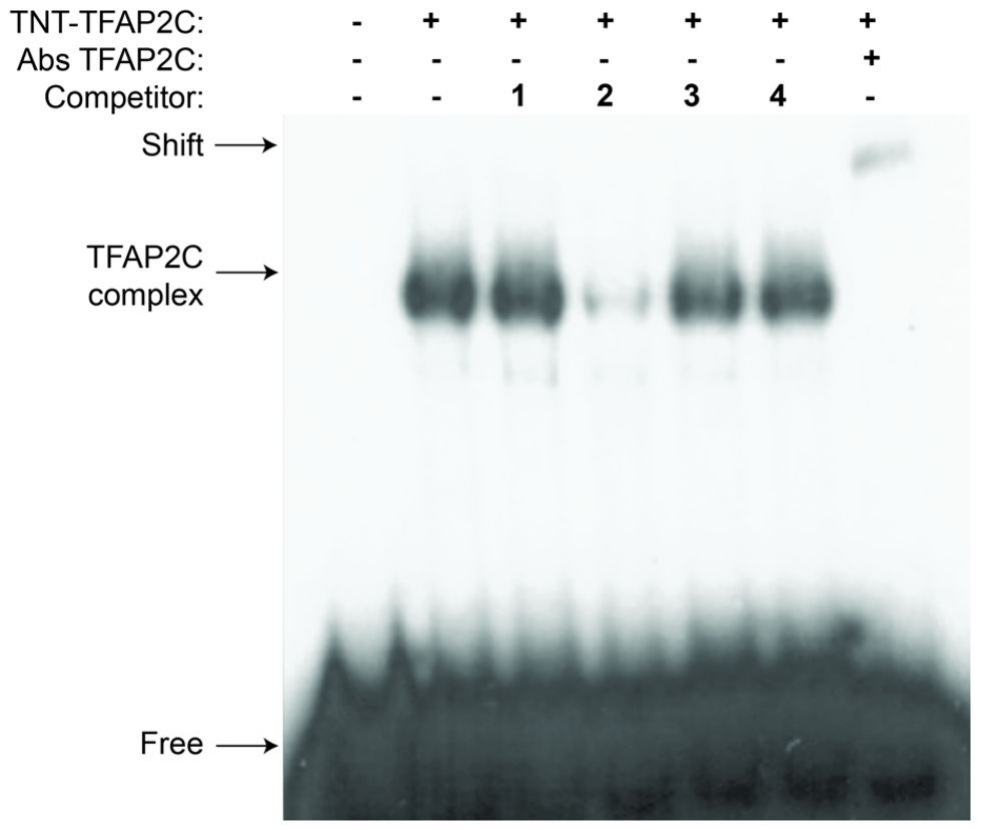
Gel-shift analysis of TFAP2C. The figure shows TFAP2C interactions with 32P-labeled promoter region of ECM1, consecutive cold competitors (F1-4) and Abs against TFAP2C used in super-shift performance. The sequences are as follows: F1 – ATCAGTCCCATTCTTCTCAATCCTCTGA;. F2- GGA**GGCTTCAGGG**TGACTTTGGGAGGAGTGGCTT C C A G A G G AGGAGCAGCTGGGAC;. F3 – TGAGTCATGGCAGGAAGCTGAGGAGGGC and. F4 - GGGAGATCACACCAGACAATTATAAAAG. In F2, the underlined sequence corresponds to the site identified by ChIP-seq and the bold sequence corresponds to the upstream site that partially competes in the assay.

In order to confirm the direct binding of TFAP2C with the promoter region of ECM1 predicted by ChIP-seq assay, a gel shift assay (GSA) was performed. The sequence of the promoter region of ECM1 localized under the peak was chosen as a probe in GSA to determine exact binding site. The fragment containing the binding site identified by ChIPseq did not appear to compete effectively in this assay, However, an upstream fragment (by 20bp) containing a potential TFAP2C binding site (GCTTCAGGG) did appear to partially compete, although mutation of this site did not alter promoter activity in our luciferase assay (data not shown). Since the sites are close together, the GSA was repeated using a fragment containing both sites. As shown in Figure **6**, the new fragment (ECM1_F2) competes more effectively. Taken together, the data suggests that both these sites may be important for the stability of TFAP2C binding along the chromatin in vivo. Addition of TFAP2C antibody results in a supershift, confirming the origin of the gel shift complex.

### ECM1 affected cell adhesion

**Figure 7:**
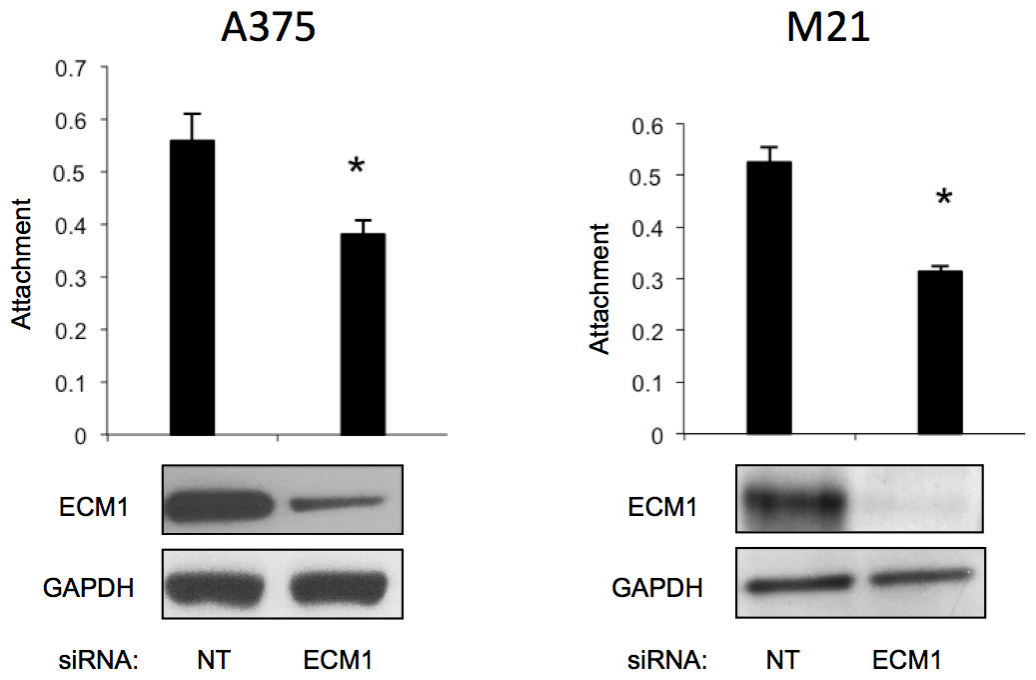
ECM1 silenced cells, as evaluated by western blots, showed significantly reduced cell attachment (*p<0.01) for both A375 and M21 cell lines when compared to cells transfected with NT siRNA. Attachment was measured as described in the methods section.

Downregulation of ECM1 did not appear to affect cell migration or invasion in either of the melanoma cell lines (data not shown). In contrast, cell adhesion as evaluated by attachment to a plastic surface was affected in both cell lines examined. As seen in Figure 7, silencing of ECM1 led to significantly reduced cell A375 and M21 cell attachment (P<0.01) when compared to cells transfected with non-targeting siRNA.

## Discussion

ECM1 over-expression has been associated with a poor prognosis in multiple malignancies; however, knowledge regarding the mechanisms affecting this is limited. In addition, while ECM1 expression has been evaluated in several epithelial malignancies, the status of ECM1 in melanocyte-derived tumors is not known. To the best of our knowledge, we are the first group to evaluate this and show that ECM1 is also over-expressed in selected melanoma cell lines, when compared to primary melanocytes.

TFAP2A is a well-recognized tumor suppressor in many tumor types via its activation of p21 Waf1/Cip1 expression, which leads to cell cycle arrest (reviewed in 23). In addition, TFAP2A is a putative p53 binding partner and has been shown to stimulate p53 transcription. Loss of TFAP2A expression is associated with progression of human melanoma [1]. Studies show that inactivation of TFAP2A via a dominant negative mechanism augments the tumorigenicity of non-metastatic melanoma cell lines in nude mice. Furthermore, enhanced over-expression of TFAP2A in metastatic melanoma cells inhibits tumor progression at cutaneous sites and reduces the formation of lung metastases after intravenous injection. Lastly, primary melanomas with reduced nuclear TFAP2 expression are associated with aggressive clinicopathologic features and shorter recurrence free survival [24].

The role of TFAP2C in carcinogenesis is less well studied. In contrast to TFAP2A, TFAP2C has been shown to be over-expressed, rather than lost in breast cancer cell lines and primary tumors [25]. Some studies show that TFAP2C expression induces *p21* mRNA and protein expression, leads to cell cycle arrest and inhibits the growth of human breast cancer cells [26]. TFAP2C is also over-expressed in primary ovarian tumors [27], with increased expression reported in higher stage tumors. Primary testicular tumors also overexpress TFAP2C [28] and its expression has potential utility for the detection of carcinoma-in-situ in that malignancy [29]. In contrast to TFAP2A, there is very limited data regarding the role of TFAP2C in melanoma. Penna et al. [30] recently described a microRNA miR-214 controlled pathway that regulates metastatic ability via direct action on TFAP2C expression, although it also indirectly regulates TFAP2A. However, it is important to point out that both TFAP2A and TFAP2C have been reported to have dual roles in carcinogenesis, i.e. either inhibitory or promoting depending on the specific tissues and stages of cancer progression. These discrepancies may also be related to varying methods of evaluating transcription factor expression [23].

Since TFAP2A and C have been reported as possible upstream regulators of ECM1 expression in prior studies from our lab [31] and TFAP2 is an important regulator of melanoma pathogenesis, we also examined expression of these AP2 factors in the melanoma cell lines that were evaluated for ECM1 expression.

We found that ECM1 expression paralleled TFAP2C expression levels in many, but not all of the cell lines examined. The role of TFAP2C in regulating ECM1 expression was further confirmed in the experiments modulating TFAP2 expression, as upregulation of TFAP2C and TFAP2A led to increased ECM1 expression. Since ECM1 levels appeared to have an inverse correlation with TFAP2A expression (in contrast to that observed in HMEC’s) and knowledge regarding the role of TFAP2C in melanomas is rather limited, we focused additional investigations on this association. As expected, knockdown of TFAP2C led to a reduction in ECM1 expression, thus lending further support to the hypothesis that ECM1 expression is regulated by TFAP2C. Our observation that TFAP2A overexpression also seemed to affect ECM1 expression may be related to the supraphysiologic levels of TFAP2A attained with adenoviral infection. It may also be secondary to the fact that TFAP2 family member are able to regulate each other transcriptionally [32] and TFAP2C is known to directly affect TFAP2A expression, at least in part [30]. Nonetheless, ours is the first report examining the effect of TFAP2 modulation on ECM1 expression levels.

We have also characterized the human *ECM1* promoter and shown that an approximately 100 bp fragment upstream of the transcription start site is sufficient for luciferase expression in A375 cell line. This region also contains a TATA box and binding sites for AP-1, Sp-1 and Ets family of transcription factors, which are also conserved in the mouse Ecm1 promoter [21]. Analyses by TESS (Transcription Element Search System) and Genomatix (Ann Arbor, MI) did not identify an obvious TFAP2 binding site in this “minimum promoter” area, however ChIP-seq identified a binding site in this region, which was confirmed by gelshift. The abrogation of luciferase reporter activity when this binding site was mutated indicates the importance of this sequence for AP2C binding. No peaks were noted for TFAP2C in these regions in MCF7 cells (unpublished data) that are ECM1 negative, but express abundant TFAP2C. Gelshift assays further confirm binding of TFAP2C in this region.

To summarize, our studies show that TFAP2C is an important player in the regulation of ECM1 expression in melanoma cells and that its effect is mediated by direct interaction with the ECM1 promoter. Our data have several important biological implications. The AP2 family of transcription factors is known to play an important role in the differentiation and survival of melanocytes. Additionally, multiple TFAP2 factors can function redundantly to promote melanocyte differentiation [33]. While TFAP2A has been extensively studied in melanoma, our investigations show an as yet unrecognized and understudied role for TFAP2C in this malignancy via its regulation of ECM1 expression. This is not entirely unexpected, as many TFAP2 family members have similar DNA-binding specificity [31]. Although ECM1 is overexpressed in a number of epithelial malignancies and typically associated with a worse prognosis, not much is known about the mechanism of this effect. A possible role for ECM1 in promoting angiogenesis was initially suggested by *in-situ* hybridization studies of mouse embryos indicating that ECM1 was associated with blood vessels and that its expression pattern was similar to that of flk-1, a recognized marker for endothelium. More direct evidence for the role of ECM1 in angiogenesis came from the observation that highly purified recombinant ECM1 stimulated the proliferation of cultured endothelial cells and promoted blood vessel formation in the chorioallantoic membrane of chicken embryos [11]. Sercu et al. [5] postulated that the binding of ECM1 to components of the dermal-epidermal junction *in vivo* may act as a “biological glue” in the framework of normal skin where it is highly expressed. Since tumor cell invasion has been described to include several steps such as cell adhesion to the extracellular matrix, degradation of the extracellular matrix components and tumor cell motility followed by cell detachment [34], one might speculate that altered expression of this “glue” may alter tumor cell attachment and invasion and thus affect metastatic potential and prognosis. Indeed, recent studies in a cholangiocarcinoma cell line show that ECM1 downregulation results in reduced migration and invasiveness in *in vitro* studies [13]. . While we did not note alterations in these characteristics in the melanoma lines examined, ECM1 downregulation does appear to significantly affect cell attachment. Ours is the first study to report the effects of ECM1 on this cellular characteristic and future studies will need to address the mechanisms responsible for this effect. In any case, given the above TFAP2C may also be an important mediator of melanoma prognosis (similar to TFAP2A) via its effects on ECM1 and other genes. Additional studies will be needed to 1) evaluate the expression of ECM1 in primary melanomas and discern whether it is an important determinant of prognosis in patients with these tumors; and 2) assess the viability of targeting ECM1expression as a potential therapeutic strategy for the treatment of melanoma
